# 二氧化硅微球精准制备最新研究亮点

**DOI:** 10.3724/SP.J.1123.2024.11003

**Published:** 2024-12-08

**Authors:** Quan BAI

**Affiliations:** 合成与天然功能分子教育部重点实验室,西北大学现代分离科学研究所,陕西省现代分离科学重点实验室,西北大学化学与材料科学学院,陕西 西安 710127

高效液相色谱(HPLC)被广泛用于生物、化工、制药、食品分析、环境保护等领域,是复杂样品分离分析的重要工具。众所周知,色谱分离的核心是色谱填料。二氧化硅微球具有机械强度高、比表面积大和表面易于修饰等优点,已成为应用最广泛的色谱填料基质。目前,高性能的二氧化硅微球大多依赖进口,价格昂贵,且其制备技术多为商业机密。因此,开发简单、经济的HPLC级二氧化硅微球成为研究热点。

根据van Deemter方程,色谱柱的柱效取决于微球的粒径大小、粒径分布、比表面积、孔道结构及表面功能基团。因此,粒径与孔结构的精准控制是实现高效色谱分离的关键。在过去几十年中,色谱学家主要通过减小微球的粒径来实现更高的分离效率。然而,小粒径填料带来的背压显著升高、摩擦热问题无法被忽视,加之色谱柱的装填难度亦显著增大,因此亚2 μm以下的填料仍然难以被全面接受。现代色谱工业中常用的微球制备方法主要有喷雾干燥法、聚合诱导胶体团聚法和溶胶-凝胶法等。所制备的硅胶微球存在单分散性差、粒径分布宽、孔径难以控制、金属含量超标、需要分级处理等问题。制备过程不仅导致大量化学材料的浪费,还会对环境造成潜在的污染。在传统合成方法中,微球的孔结构往往会受粒径与形貌的影响,这使得孔结构的调控变得困难,同时也导致可实现的孔道尺寸范围十分有限。截至目前,精确制备硅胶微球仍然是一个巨大挑战。因此,发展一种针对不同粒径、孔结构和材料化学的球形色谱材料的通用合成策略是开发下一代高效色谱材料的关键。

针对上述难题,近日,厦门大学化学化工学院张博课题组开发了一种基于液滴微流体技术的精准制备策略,用于制备高性能的单分散硅胶微球,并具有对微球的颗粒形态、孔结构和材料化学分别进行独立调控的能力^[1]^。该策略使用以聚二甲基硅氧烷(PDMS)为基材的光刻芯片作为液滴生成平台,单张芯片内集成了120通道并行的流式聚焦液滴生成装置,能以高达240 kHz/chip的超高通量制备尺寸均一的液滴。液滴经过孵育、清洗和煅烧程序后,固化为硅胶微球。通过液滴合成策略制备的微球具有极窄的粒径分布(相对标准偏差<3%)和极高的转化率(近100%)。与现有的商品化填料相比,这种单分散微球展现出优异的分离效率。更重要的是,相比传统方法,液滴合成策略在微球粒径、孔结构、材料化学控制等方面具有显著优势。通过调节连续相与分散相的流速,液滴合成策略能够在保持单分散性的前提下自由调节微球粒径。此外,通过调节致孔剂的相对分子质量和含量,液滴合成策略能够实现无孔、介孔、大孔甚至灌流孔微球的可控制备。这种连续的孔调节能力可用于蛋白质分离的选择性精细调节,从而实现高分辨蛋白质色谱分离。最后,除了经典的二氧化硅微球,通过改变单体类型,杂化硅胶(乙基桥联、苯基桥联)和过渡金属氧化物(ZrO_2_和TiO_2_)等材质的单分散微球均可通过液滴合成策略制备,并广泛适用于小分子药物、磷酸腺苷、糖基化/磷酸化蛋白与多肽等对象的分离。

该方法的优越性主要体现在4个方面:(1)全制备过程实现近100%的转化效率,避免了筛选过程造成的浪费和污染,是绿色的化学工艺;(2)对微球粒径、孔结构和材料化学的独立调控能力,避免了各化学过程之间的相互干扰,简化了合成策略;(3)对微球粒径、孔结构、材料化学的调控能力,不仅拓宽了适用范围,还可实现色谱分离过程的精细操控;(4)精准制备的色谱微球在分离效率、选择性和材料多样性等方面均展现出优异的性能。

该工作面向高效色谱材料的制备问题,基于液滴微流体技术,成功实现了硅胶微球的精准制备、高通量合成和性能精细调控,为下一代色谱材料的绿色、精准制备奠定了基础。
